# Calprotectin strongly and independently predicts relapse in rheumatoid arthritis and polyarticular psoriatic arthritis patients treated with tumor necrosis factor inhibitors: a 1-year prospective cohort study

**DOI:** 10.1186/s13075-018-1764-z

**Published:** 2018-12-13

**Authors:** José Inciarte-Mundo, Julio Ramirez, Maria Victoria Hernández, Virginia Ruiz-Esquide, Andrea Cuervo, Sonia Raquel Cabrera-Villalba, Mariona Pascal, Jordi Yagüe, Juan D. Cañete, Raimon Sanmarti

**Affiliations:** 1 Department of Rheumatology, Hospital Clinic, University of Barcelona, Carrer Villarroel 170, 08036 Barcelona, Spain; 2 Department of Immunology, Hospital Clinic, University of Barcelona, Barcelona, Spain

**Keywords:** Calprotectin, TNFi serum trough levels, Ultrasound, Relapse, Biomarkers, Predictors, Rheumatoid arthritis, Psoriatic arthritis

## Abstract

**Background:**

Calprotectin is a biomarker of disease activity in rheumatoid arthritis (RA) and psoriatic arthritis (PsA) and predicts relapse in juvenile idiopathic arthritis. Higher drug trough serum levels are associated with a good response in patients treated with tumor necrosis factor inhibitors (TNFi). Power Doppler ultrasound synovitis is predictive of relapse and structural damage progression in patients in clinical remission. The purpose of this study was to analyze the accuracy of serum calprotectin levels, drug trough serum levels (TSL), and power Doppler (PD) activity as predictors of relapse in RA and PsA patients in remission or with low disease activity receiving TNFi.

**Methods:**

This was a longitudinal, prospective, 1-year single-center study of 103 patients (47 RA, 56 PsA) receiving TNFi in remission or with low disease activity (28-joint Disease Activity Score (DAS28) ≤ 3.2). The predictive value of serum calprotectin, TNFi TSL, and PD were assessed using receiver operating characteristic (ROC) analyses. To illustrate the predictive performance of calprotectin, TNFi TSL, and PD score, Kaplan-Meier curves were constructed from baseline to relapse. Associations between baseline factors and relapse were determined using Cox regression models. Multivariate models were constructed to analyze the effect of covariates and to fully adjust the association between calprotectin, TNFi TSL, and PD score with relapse. A generalized estimating equation model with an identity link for longitudinal continuous outcomes was used to assess the effect of covariates on TNFi TSL.

**Results:**

Ninety-five patients completed 1 year of follow-up, of whom 12 experienced a relapse. At baseline, relapsers had higher calprotectin levels, lower TNFi TSL, and higher PD activity than nonrelapsers. ROC analysis showed calprotectin fully predicted relapse (area under the curve (AUC) = 1.00). TNFi TSL and PD had an AUC of 0.790 (95% confidence interval (CI) 0.691–0.889) and 0.877 (95% CI 0.772–0.981), respectively. Survival analyses and log rank tests showed significant differences between groups according to calprotectin serum levels (*p* < 0.001), TNFi TSL (*p* = 0.004), and PD score (*p* < 0.001). Univariate Cox regression models showed that time-to-remission/low disease activity (hazard ratio (HR) = 1.17, *p* < 0.001), calprotectin levels (HR = 2.38, *p* < 0.001), TNFi TSL (HR = 0.47, *p* = 0.018), and PD score (HR = 1.31, *p* < 0.001) were significantly associated with disease relapse. In the multivariate analysis, only baseline calprotectin levels independently predicted disease relapse (HR = 2.41, *p* = 0.002). The generalized estimating equation analysis showed that only disease activity by DAS28-erythrocyte sedimentation rate (ESR) was significantly associated with longitudinal changes in TNFi TSL (regression coefficient 0.26 (0.0676 to 0.0036), *p* = 0.001).

**Conclusion:**

Time-to-remission/low disease activity, calprotectin serum levels, TNFi TSL, and PD score were significantly associated with disease relapse. However, only baseline calprotectin serum levels independently predicted disease relapse in RA and PsA patients under TNFi therapy.

## Background

Biological therapies have dramatically improved the management and prognosis of rheumatoid arthritis (RA) and psoriatic arthritis (PsA). Remission or low disease activity is possible in an increasing number of patients. Nevertheless, persistent remission is more difficult to achieve since disease relapses are common. In patients classified as in clinical remission, low-grade inflammatory activity may persist but remain undetectable by routine tests. Subclinical disease activity may result in disease relapse. Musculoskeletal ultrasound (MSUS) has shown that a significant proportion of patients classified as in remission exhibit a power Doppler (PD) signal [1] which is associated with short-term relapse in RA patients [2, 3].

Calprotectin, a member of the S100 protein family, is an important proinflammatory factor of innate immunity, acting as an endogenous damage-associated molecular pattern molecule via Toll-like receptor 4 activation. Higher calprotectin levels have been found in serum from RA and PsA patients [4, 5]. Calprotectin serum levels correlate with disease activity and are independently associated with radiographic progression in RA [6, 7]. Moreover, calprotectin levels predict the response to biologic therapy in RA and PsA patients [8, 9]. Our research group has demonstrated that serum calprotectin stratifies disease activity more accurately than C-reactive protein (CRP) or the erythrocyte sedimentation rate (ESR) in patients receiving tumor necrosis factor inhibitors (TNFi) [10] or tocilizumab [11], and strongly correlates with power Doppler ultrasound synovitis (PDUS) in RA and PsA patients with low disease activity [12]. In other rheumatic diseases, such as juvenile idiopathic arthritis, calprotectin more accurately predicted relapse in patients in remission [13]; therefore, calprotectin might be useful in predicting relapse in RA and PsA patients with low disease activity.

Recent studies have found a significant dose-response relationship between the extent of clinical improvement and TNFi trough serum levels in RA and spondyloarthritis (SpA) patients [14, 15]. Higher TNFi trough serum levels have been associated with good therapeutic responses [16].Therefore, patients with low TNFi trough serum levels might be more prone to relapse.

The aim of this study was to determine the accuracy of baseline calprotectin, TNFi trough serum levels, and power Doppler (PD) score in predicting disease relapse in RA and PsA patients in remission or with low disease activity. In addition, we examined other baseline factors related to relapse, the performance of calprotectin and TNFi trough serum levels during relapse, and factors associated with longitudinal changes in TNFi trough serum levels.

## Patients and methods

This longitudinal prospective observational single-center study included 103 consecutive RA patients (according to American College of Rheumatology (ACR) 1987 criteria) and polyarticular PsA patients (according to the Classification of Psoriatic Arthritis (CASPAR)) from our Arthritis Unit. Patients were enrolled between March 2013 and September 2014, with follow-up ending in September 2015. All patients were in remission (28-joint Disease Activity Score (DAS28)-ESR ≤ 2.6) or had low disease activity (DAS28-ESR ≤ 3.2) in two consecutive visits at least 3 months apart and were currently receiving adalimumab (ADA), etanercept (ETN), or infliximab (IFX). Exclusion criteria included patients with higher levels of disease activity (DAS28 > 3.2) or PsA patients with axial or entheseal involvement, or an oligoarticular peripheral pattern. Details on demographic data, disease duration, autoantibody status, radiological data, concomitant conventional synthetic disease-modifying antirheumatic drug (csDMARD) therapy, and dose and duration of biological therapy were collected. Some patients received a reduced dose of biologic therapy due to persistent remission/low disease activity. A reduced dose was defined as treatment with a lower amount of the drug or longer intervals of administration than recommended in each product package insert.

### Definition of disease relapse

A disease relapse was defined using the revised European League Against Rheumatism (EULAR) response criteria [17]. The criterion for disease relapse was current DAS28 > 3.2 and an increase in DAS28 > 0.6 compared with baseline. Patients were encouraged to contact investigators if they had a disease relapse between two visits. Patients fulfilling this criterion were classified as relapsers, while patients remaining stable during follow-up were classified as nonrelapsers.

### Measurement of clinical disease activity

All patients underwent clinical assessment at baseline and at 4, 8, and 12 months, including 28-joint swollen and tender joint counts (28-SJC and 28-TJC, respectively), physician and patient global assessment with visual analogue scales (0–100 mm), ESR (mm), and CRP (mg/dL). Three composite disease activity indices were subsequently calculated: DAS28-ESR [18], Clinical Disease Activity Index (CDAI) [19], and Simple Disease Activity Index (SDAI) [20].

### Serum biomarkers: calprotectin and TNFi trough levels

Calprotectin serum levels, TNFi trough serum levels, and antidrug antibodies were determined at baseline (visit 0) and during disease relapse using an enzyme-linked immunosorbent assay (ELISA) test kit (Calprolab™ calprotectin ELISA (ALP), Calpro AS, Oslo, Norway, and Promonitor®, Progenika SA, Spain, respectively) according to the manufacturers’ protocol as previously described [10]. These ELISA kits are validated and show adequate correlation for the measurement of drug levels and antidrug antibodies [21]. To reduce variations in calprotectin determinations, the whole procedure was performed in a Triturus® autoanalyzer. Additionally, serum samples were collected at 4, 8, and 12 months of follow-up to assess longitudinal changes in drug trough serum levels.

### Imaging Biomarkers: power Doppler score

A sonographic assessment was made at baseline (visit 0) using high-sensitivity ultrasound equipment (MyLab Twice®, Esaote, Italy) as previously described [12]. Joint MSUS findings were defined according to published Outcome Measures in Rheumatology (OMERACT) definitions [22]. One experienced sonographer (JR) who was blinded to the results of the clinical joint examination evaluated 11 joints and tendons of each hand (including the proximal interphalangeal joints, metacarpophalangeal joints, and wrists) for synovial hypertrophy (SH) and intra-articular PD signal according to EULAR guidelines [23]. SH and the PD signals were graded according to the methodology of Szkudlarek et al. [24]. Intra-rater agreement on the MSUS assessment, calculated as previously described [25], was 0.83 for SH and 0.90 for PD. We made a double ultrasound assessment in the first 10 patients included in the study. The two evaluations were separated by between 24 and 72 h. The same sonographer made both ultrasound explorations and noted the results. This index was calculated as the percentage of agreement between these scores at two time points. The following cut-off values, analogous to kappa coefficients, were defined for intra-rater reliability: < 0.0 = none, 0 to 0.20 = poor, 0.21 to 0.40 = modest, 0.41 to 0.60 = fair, 0.61 to 0.80 = good, and 0.81 to 1.00 = excellent.

By summing the scores for elementary lesions in each joint, we calculated the PD score (range 0–66), the SH score (range 0–66), and the global score (which is the sum of the PD and SH scores; range 0–132). PDUS was defined as a PD signal in synovial tissue [26]. A more-stringent definition of active synovitis (ultrasound-defined active synovitis (UdAS)) developed by our group (SH grade ≥ 2 plus PDUS signal) was also recorded [25].

This study was conducted in accordance with the Declaration of Helsinki and was approved by the Clinical Research Ethics Committee of the Hospital Clínic of Barcelona (Reg. 2013/8382). Signed informed consent was obtained from all patients before study enrolment.

### Statistical analysis

Continuous data are presented as medians and interquartile ranges (IQR) and categorical variables as absolute frequencies and percentages. Relapsers versus nonrelapsers were compared using the Student’s *t* test or Mann-Whitney test when appropriate. The predictive value of calprotectin, TNFi trough serum levels, and PD score for the risk of relapse was assessed using the receiver operating characteristic (ROC), and the most sensitive and specific cut-off was identified; they were then dichotomized, applying an optimal cut-off as per ROC analysis. The predictive values, accuracy, positive likelihood ratio, and maximum Youden index were calculated. The area under the curve (AUC) was estimated using Hanley’s corrected confidence intervals (CIs). To illustrate the predictive performance of calprotectin, TNF serum levels, and PD score, Kaplan-Meier curves were constructed from baseline to relapse. Associations between baseline factors and disease relapse were assessed using Cox proportional hazards regression models. Crude odds ratios (ORs) with 95% CIs were calculated. Multivariate models were constructed to analyze the effect of covariates and to fully adjust the association between calprotectin, TNFi trough serum levels, and PD score with relapse. Models were fitted separately and compared using Akaike Information Criterion (AIC) and the Bayesian Information Criterion (BIC). The generalized estimating equation (GEE) model with an identity link for longitudinal continuous outcomes was used to assess the effect of covariates on TNFi trough serum levels at 0, 4, 8, and 12 months. The analysis was made using STATA version 11 (STATA Corp., College Station, TX, USA).

## Results

### Baseline characteristics

Of the 103 consecutive enrolled patients (47 RA, 56 PsA), eight were lost to follow-up, and 95 patients completed a 1-year follow-up (44 RA, 51 PsA). Table 1 shows the clinical characteristics at baseline. Patients included were mostly women with established disease on prolonged biological treatment: 44 patients were treated with ETN, 34 with ADA, and 17 with IFX, and 45 patients had received a reduced dose of biologics and 45 were on monotherapy. Seventy-two (75.8%) and 23 patients (24.2%) fulfilled the DAS28 remission and low disease activity criteria, respectively. Fifty (52.6%) patients had PDUS, and the median number of joints with PDUS was 1. Twenty-nine (30.5%) patients fulfilled UdAS criteria.

**Table 1:** Baseline characteristics of patients with disease relapse (“relapsers”) or stable disease activity (“nonrelapsers”) during 1 year of follow-up

Characteristic	Total (*n* = 95)	Nonrelapsers (*n* = 83)	Relapsers (*n* = 12)	*p* value
Age (years)	57 (50–66)	57 (50–66)	57 (48–63.5)	0.614
Female, *n* (%)	61 (64.2%)	53 (63.9%)	8 (66.7%)	1.000
Disease duration (years)	15 (9–21)	15 (9–21)	14.5 (7.5–24.5)	0.831
Diagnosis, *n* (%)				0.215
Psoriatic arthritis	51 (53,7%)	47 (56.6%)	4 (33.3%)	
Rheumatoid arthritis	44 (46.3%)	36 (43,4%)	8 (66.7%)	
Time to csDMARD (months)	25.6 (5.1–62.2)	24.4 (5.5–62.2)	32.6 (5.1–92.3)	0.911
Time to bDMARD (months)	98.5 (36.9–165.9)	98.5 (38.8–160.9)	95.9 (33.6225.9)	0.823
Time-to-remission/LDA (months)	3.27 (2.13–4.3)	3.07 (1.9–3.97)	20.4 (16.8–24.3)	**< 0.001**
Time-in-remission/LDA (months)	58.7 (26.7–86.6)	60.1 (27.6–88.0)	25.0 (9.4–59.3)	**0.027**
Calprotectin (μg/mL)	1.66 (0.69–2.68)	1.44 (0.62–2.34)	6.01 (5.01–6.44)	**< 0.001**
CRP (mg/dL)	0.10 (0.04–0.26)	0.09 (0.03–0.22)	0.17 (0.04–0.52)	0.388
ESR (mm)	10 (7–18)	10 (7–16)	14.5 (8–21.5)	0.225
Albumin (g/L)	42 (31–48)	43 (31–48)	31 (31–47)	0.210
Biologic treatment, *n* (%)				0.843
Adalimumab	34 (35.8%)	30 (36.1%)	4 (33.3%)	
Etanercept	44 (46.3%)	39 (47.0%)	5 (41.7%)	
Infliximab	17 (17.9%)	14 (16.9%)	3 (25.0%)	
Biological treatment duration (months)	61.6 (30.8–91.4)	63.2 (31.8–92.7)	39.9 (25.1–61.2)	0.136
Reduced dose of biologics^a^, *n* (%)	45 (47.4%)	40 (48.2%)	5 (41.7%)	0.672
Monotherapy, *n* (%)	45 (47.4%)	42 (50.6%)	3 (25.0%)	0.127
Concomitant steroids, *n* (%)	18 (18.9%)	13 (15.7%)	5 (41.7%)	**0.047**
Global TNFi trough serum levels (μg/mL)	2.20 (1.07–6.26)	2.70 (1.18–6.83)	1.14 (0.73–1.52)	**0.001**
Adalimumab (μg/mL)	7.04 (2.69–9.40)	7.19 (5.78–9.88)	1.28 (0.73–1.59)	**0.009**
Etanercept (μg/mL)	1.45 (1.00–2.25)	1.52 (0.92–2.32)	1.24 (1.20–1.54)	0.698
Infliximab (μg/mL)	2.94 (0.86–3.26)	3.16 (1.92–4.84)	0 (0–1.05)	**0.023**
DAS28-ESR	2.03 (1.68–2.6)	1.99 (1.67–2.52)	2.43 (1.89–2.74)	0.145
Remission, *n* (%)	72 (75.8%)	65 (78.3%)	7 (58.3%)	0.175
Low disease activity, *n* (%)	23 (24.2%)	18 (21.7%)	5 (41.7%)	0.127
SDAI	6.03 (2.2–6.26)	6.02 (2.11–6.22)	6.18 (4.53–6.96)	0.087
CDAI	6 (2–6)	6 (2–6)	6 (4–6.5)	0.220
Global ultrasound score	8 (3–13)	8 (3–13)	17.5 (9.5–31)	**0.041**
PD score	1 (0–2)	1 (0–2)	5.5 (2.5–10)	**< 0.001**
SH score	7 (3–11)	6 (3–10)	8.5 (5–12)	0.210
PDUS, *n* (%)	50 (52.6%)	38 (45.8%)	8 (66.6%)	**< 0.001**
Number of joints with PDUS	1 (0–2)	1 (0–2)	3.5 (2.5–5)	**< 0.001**
UdAS, *n* (%)	29 (30.5%)	17 (20.5%)	7 (58.3%)	**< 0.001**

Values are shown as median (interquartile range) unless otherwise stated

Significant *p* values are shown in bold typeface

*bDMARD* biologic disease-modifying antirheumatic drug, *CDAI* Clinical Disease Activity Index, *CRP* C-reactive protein, *csDMARD* conventional synthetic disease-modifying antirheumatic drug, *DAS28* 28-joint Disease Activity Score, *ESR* erythrocyte sedimentation rate, *LDA* low disease activity, *PD* power Doppler, *PDUS* power Doppler ultrasound synovitis, *SH* synovial hypertrophy, *SDAI* Simplified Disease Activity Index, *TNFi* tumor necrosis factor inhibitors, *UdAS* ultrasound-defined active synovitis

^a^Treatment regimen with a lower amount of the drug or longer intervals of administration than those recommended in the package insert for each product

### Baseline serum calprotectin, TNFi trough serum levels, and PDUS, and association with disease relapse

Eighty-three patients remained in remission/low disease activity over 1 year, and 12 (eight RA, four PsA) relapsed (six patients at 4 months, four at 8 months, and two at 12 months). No differences in disease duration, biological treatment duration, reduced dose, or monotherapy were observed between relapsers and nonrelapsers (Table 1).

Clinically, relapsers had a longer time-to-remission/low disease activity, shorter time-in-remission/low disease activity, and were more often on steroid treatment compared with nonrelapsers. No differences in time to csDMARD or biological DMARD were observed (Table 1).

At baseline, relapsers had higher calprotectin levels and lower mean TNFi trough serum levels, even when analyzed according to biological agent in the ADA and IFX group. No differences in CRP or ESR were observed (Table 1).

Relapsers showed higher PD activity, with more patients with PDUS, more joints with a PD signal, and a higher global score and PD score than nonrelapsers. In addition, more relapsers fulfilled UdAS criteria. Similar results were obtained when patients were analyzed according to diagnosis (Table 1).

#### Calprotectin had greater discriminatory capacity to predict relapse than TNFi trough serum levels and the PD score

The cut-off levels determined by ROC analysis for the optimal prediction of relapse for calprotectin, TNFi serum levels, and PD score were 3.7 μg/mL, 1.61 μg/mL, and 4, respectively, and the Youden index was 0.99, 0.51, and 0.64, respectively. ROC analyses showed that calprotectin (AUC = 1, 95% CI 1.00–1.00) had greater discriminatory capacity than TNFi trough serum levels and PD score in predicting relapse. Table 2 shows further diagnostic statistics of the dichotomized biomarkers. This calprotectin cut-off level had a sensitivity of 100% and a specificity of 98.8% for the diagnosis of a relapse (positive likelihood ratio 83, negative likelihood ratio 0).

**Table 2:** Diagnostic statistics of dichotomized biomarkers

	Calprotectin	TNFi trough serum levels	Power Doppler score
Cut-off level to predict relapse	≥ 3.7 μg/mL	< 1.61 μg/mL	≥ 4
Area under the curve	1.000 (1.000–1.000)	0.790 (0.691–0.889)	0.877 (0.772–0.981)
Sensitivity	100%	68.7%	75.0%
Specificity	98.8%	83.3%	89.2%
Positive likelihood ratio	83.0	4.12	6.92
Negative likelihood ratio	0.00	0.37	0.28
Correctly classified	98.9%	70.5%	87.4%
Youden index	0.99	0.51	0.64

*TNFi* tumor necrosis factor inhibitors

#### Predictive performance of calprotectin, TNFi trough serum levels, and PD score for disease relapse

**Fig. 1:**
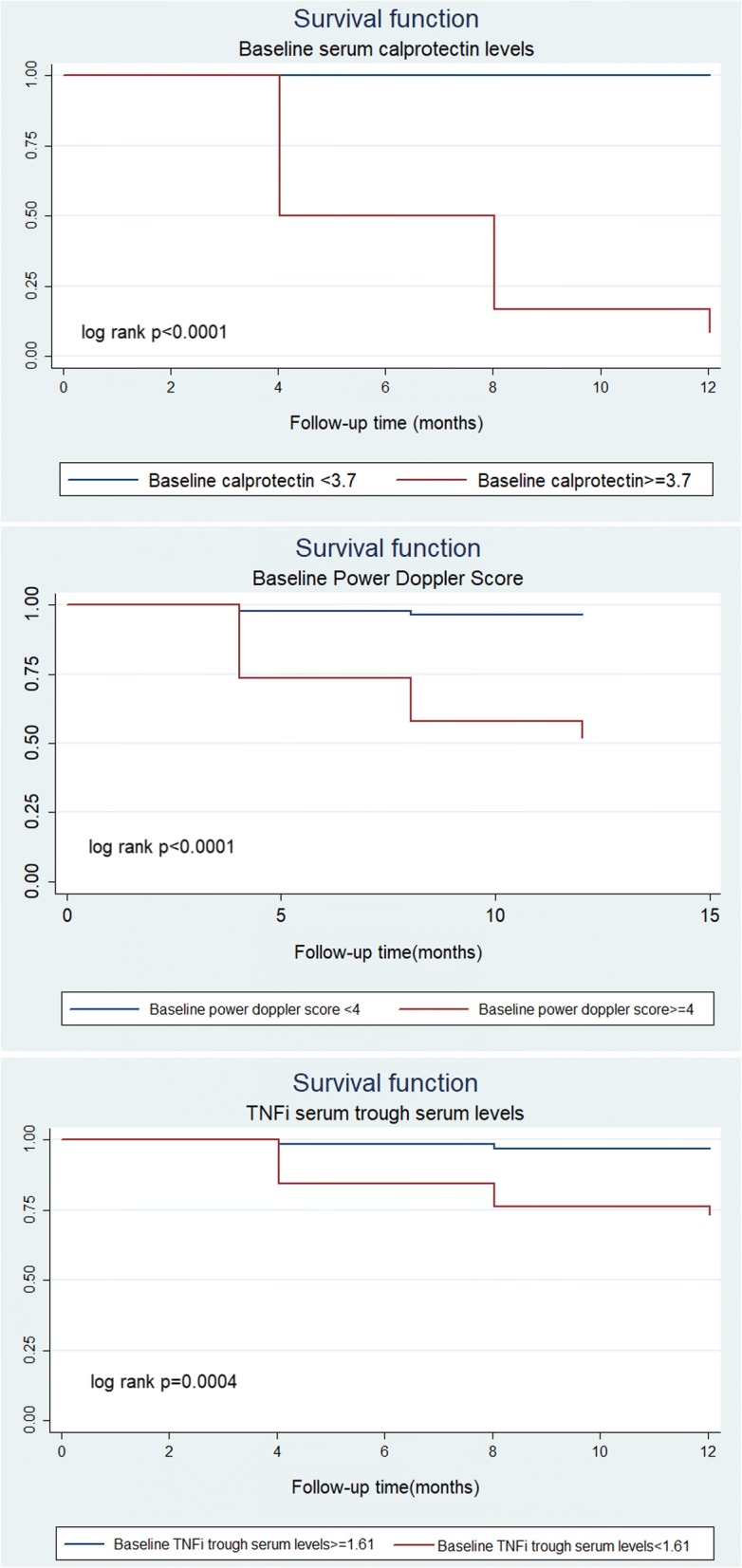
Kaplan-Meier curves of biomarkers from the time of inclusion to disease relapse. **a** Calprotectin serum levels. **b** Power Doppler score. **c** Tumor necrosis factor inhibitors (TNFi) trough serum levels

Kaplan-Meier curves were constructed from baseline to disease relapse to illustrate the predictive performance of calprotectin, TNF serum levels, and PD score. Biomarkers were dichotomized into high versus low according to cut-off levels for optimal prediction of relapse previously calculated by ROC analysis. Survival analyses by log rank tests showed significant differences between groups according to calprotectin serum levels (*p* < 0.001), TNFi trough serum levels (*p* = 0.004), and PD score (*p* < 0.001) (Fig. 1).

#### Factors associated with disease relapse

Univariate Cox regression analyses showed time-to-remission/low disease activity (hazard ratio (HR) = 1.17, *p* < 0.001) was the only clinical variable associated with an increased risk of relapse. In addition, steroid use, calprotectin, TNFi trough serum levels, and PD score were significantly associated with disease relapse. In the multivariate regression, only baseline calprotectin levels were independently associated with disease relapse (HR = 2.74, *p* < 0.0001). No significant interaction terms were detected (Table 3).

**Table 3:** Baseline factors associated with disease relapse using Cox proportional hazards regression models

Baseline characteristics	Univariate	Multivariate
	HR (95% CI; *p* value)	HR (95% CI; *p* value)
Calprotectin (μg/mL)	**2.38 (1.79–3.16; < 0.0001)**	**2.74 (1.71–4.41; < 0.0001)**
Power Doppler score, 0–66	**1.31 (1.18–1.45; < 0.0001)**	
TNFi trough serum levels (μg/mL)	**0.47 (0.26–0.88; 0.018)**	0.36 (0.10–1.30; 0.120)
DAS-ESR	2.10 (0.72–6.18; 0.175)	
Time-to-remission (months)	**1.17 (1.11–1.23; < 0.0001)**	
Time-in-remission (months)	0.98 (0.97–1.00; 0.087)	
Female gender	1.25 (0.38–4.15; 0.716)	
Disease duration (months)	1.02 (0.96–1.08; 0.510)	
Diagnosis, RA/PsA	2.39 (0.72–7.93; 0.155)	0.80 (0.10–6.30; 0.832)
Age (years)	0.98 (0.94–1.03; 0.535)	
Monotherapy	0.36 (0.10–1.33; 0.125)	0.51 (0.08–3.35; 0.484)
Steroids	**3.12 (1.02–10.1; 0.046)**	0.22 (0.04–1.21; 0.082)

Significant values are shown in bold typeface

*CI* confidence interval, *DAS-ESR* Disease Activity Score-erythrocyte sedimentation rate, *HR* hazard ratio, *PsA* psoriatic arthritis, *RA* rheumatoid arthritis, *TNFi* tumor necrosis factor inhibitors

### Calprotectin and TNFi trough serum levels during relapse

**Fig. 2:**
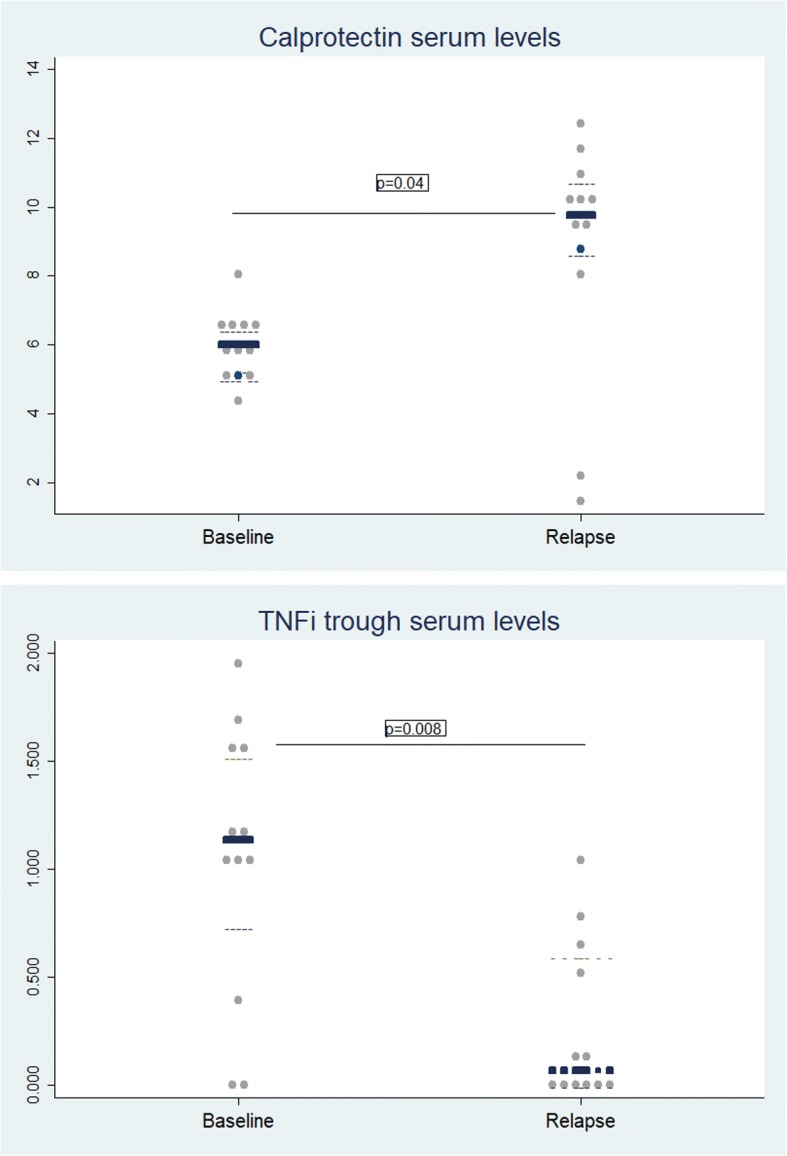
Biomarkers levels during relapse. **a** Calprotectin serum levels during relapse. **b** TNFi trough serum levels during relapse

Serum samples from the 12 relapsers were analyzed during relapse. A significant increase in calprotectin levels was observed in 10 patients, while TNFi trough serum levels decreased in all relapsers compared with baseline values (Fig. 2).

After 1 year, antidrug antibodies were detected in four patients, who all had a relapse (ADA in two out of four relapsers and IFX in two out of three relapsers). All patients with detectable antidrug antibodies had undetectable TNFi trough serum levels.

#### Longitudinal changes in TNFi trough serum levels

Multivariate GEE analysis showed that only disease activity by DAS28-ESR was significantly associated with longitudinal changes in TNFi trough serum levels (regression coefficient 0.26 (0.068 to 0.004), *p* = 0.001). This effect was independent of other factors analyzed, such as diagnosis, monotherapy, steroids, and reduced dose of biologics (Table 4).

**Table 4:** Factors associated with longitudinal changes in TNFi trough serum levels over 1 year of follow-up using GEE multivariate models

Variable	TNFi trough serum levels	*p* value
	β coefficient (95% CI)	
DAS28-ESR	**0.26 (0.068–0.004)**	**0.001**
Reduced dose (yes/no)	0.022 (0.168–0.012)	0.271
Diagnosis (RA/PsA)	0.005 (0.058–0.144)	0.662
Monotherapy (yes/no)	0.0004 (0.090–0.062)	0.863
Follow-up		
Baseline	1	
4-month visit	0.012 (0.044–0.0004)	0.023
8-month visit	0.004 (0.032–0.002)	0.280
12-month visit	0.002 (0.005–0.032)	0.409
Steroids	0.020 (0.325–0.090)	0.538
Constant	**4.45 (2.99 to 6.25)**	**< 0.0001**

Significant values are shown in bold typeface

*CI* confidence interval, *DAS28-ESR* 28-joint Disease Activity Score-erythrocyte sedimentation rate, *GEE* generalized estimating equation, *PsA* psoriatic arthritis, *RA* rheumatoid arthritis, *TNFi* tumor necrosis factor inhibitors

## Discussion

The results of our study show that TNFi trough serum levels, PD score, and time-to-remission/low disease activity were significantly associated with disease relapse. However, only baseline calprotectin levels independently predicted disease relapse in RA and PsA patients treated with TNFi after a 1-year follow-up.

The innate immune system plays a central role in local inflammation in RA and PsA patients, as demonstrated by the massive infiltration of macrophages into the synovial tissue [27–29]. Most biologics, including TNFi and anti-interleukin (IL)-6, specifically target cytokines related to this cell type, and thus it is not surprising that changes in the number of synovial sublining macrophages correlate with clinical improvement in RA patients [30]. Calprotectin is released by innate immune cells, particularly neutrophils and macrophages, and calprotectin levels provide reliable information about neutrophil activation associated with disease activity in RA and PsA patients [4, 25, 31–33].

In our cohort, calprotectin performed particularly well because all relapsers showed a baseline calprotectin level > 3.7 μg/mL; this high accuracy in predicting disease relapses is not completely unexpected. In other inflammatory conditions, such as juvenile idiopathic arthritis (JIA) [13], systemic-onset juvenile idiopathic arthritis (SoJIA) [34], ANCA-associated vasculitis [35], or inflammatory bowel disease (IBD) [36], calprotectin has been shown to be a more sensitive indicator of disease activity and, in consequence, more accurately predicts relapse [34, 37–40].

We have previously demonstrated that RA patients in remission with a PD signal had significantly greater macrophage infiltration in the synovial tissue, comparable to clinically active patients [25], which may explain why these patients had threefold higher calprotectin serum levels compared with healthy blood donors [10]. As expected, relapsers showed a significant increase in calprotectin serum levels during the disease relapse, reflecting local ongoing inflammation. Thus, calprotectin serum levels may identify residual inflammatory activity in patients with low disease activity and are a strong predictor of disease relapse.

Nonrelapsers had higher drug trough serum levels than relapsers, even according to the biologic agent, and these significantly decreased during relapse, supporting a previous report on the relationship between inflammation and TNFi pharmacokinetics in RA. In patients with substantial inflammation, IFX serum levels were decreased due to its capture by TNFα [41]. Our results support the emerging role of TNFi trough serum levels as a relevant factor associated with disease activity in RA and PsA patients receiving biological therapy [14–16, 42].

In accordance with previous reports [1], 52.6% of our patients in clinical remission or with low disease activity (65.9% RA, 41.1% PsA) showed PD activity in at least one joint, which has been associated with a higher risk of disease relapse in RA [2, 3]. Although there are no data available for polyarticular PsA, logistic regression analyses showed that the PD score, regardless of diagnosis, was associated with relapse, which is a relevant contribution of our study. Remission is not equivalent to the absence of inflammation. Persistence of the PD signal could explain the radiographic damage progression observed in RA and PsA patients in clinical remission [1, 43].

As in previous reports, the only clinical factor associated with relapse identified in our study was time-to-remission/low disease activity. Sustained remission was only determined by time-to-remission in a cohort of early RA patients; the probability of sustained remission increased significantly with decreasing time-to-remission, independently of the DMARD type or strategy [44].

Accurately predicting relapses could avoid delays and related costs [45]. Calprotectin [46] and TNFi trough levels [47] have been included in guidelines on monitoring the therapeutic response in IBD patients. In RA, there are some proposals for decision algorithms which have included calprotectin [48] and TNFi trough levels [49].

The small sample size and low percentage of relapsing patients (12.6%) could be a limitation. However, we were able to detect statically significant differences between relapsers and nonrelapsers, which is a strength of our study. We presumed that the use of DAS28 ≤ 3.2 as the inclusion criteria could limit the interpretation of our results, since this composite index allows residual tender and/or swollen joints in patients classified as being in remission. However, it is the most widely used composite articular index, and therefore our results would be clinically applicable, contributing to the strengths of this study. Although developed to assess disease activity in RA, studies support the use of DAS28 in PsA patients receiving biological therapy [50]. A single rheumatologist performed the US assessment in both hands, and therefore inter-observer reliability could not be calculated, and there is a known significant variability between observers in the assessment of synovitis using US. Also, US assessment focused on the hand could be insufficient to capture inflammatory activity of systemic diseases, such as RA and PsA. However, US examination of hands only could be sufficient to detect > 90% of RA patients in the clinic with subclinical inflammation [51].

Considering these limitations, we have demonstrated a high accuracy of baseline calprotectin serum levels in predicting relapse in patients with low levels of disease activity.

## Conclusions

In RA and PsA patients with low levels of disease activity, time-to-remission/low disease activity, calprotectin serum levels, TNFi trough serum levels, and PD score were significantly associated with disease relapse. However, only baseline calprotectin levels were independently associated with disease relapse. Calprotectin may be used to stratify disease activity more accurately in patients with low disease activity, guiding therapeutic decisions towards safer and more cost-effective strategies.

## Abbreviations

ACR: American College of Rheumatology
ADA: Adalimumab
AIC: Akaike Information Criterion
AUC: Area under the curve
BIC: Bayesian Information Criterion
CASPAR: Classification of Psoriatic Arthritis
CDAI: Clinical Disease Activity Index
CI: Confidence interval
CRP: C-reactive protein
csDMARD: Conventional synthetic disease-modifying antirheumatic drug
DAS28: 28-Joint Disease Activity Score
ESR: Erythrocyte sedimentation rate
ETN: Etanercept
EULAR: European League Against Rheumatism
GEE: Generalized estimating equation
HR: Hazard ratio
IFX: Infliximab
MSUS: Musculoskeletal ultrasound
OMERACT: Outcome Measures in Rheumatology
OR: Odds ratio
PD: Power Doppler
PDUS: Power Doppler ultrasound synovitis
PsA: Psoriatic arthritis
RA: Rheumatoid arthritis
ROC: Receiver operating characteristic
SDAI: Simple Disease Activity Index
SH: Synovial hypertrophy
TNFi: Tumor necrosis factor inhibitors
UdAS: Ultrasound-defined active synovitis
